# The Gradient Effect on Cyclic Behavior of 316L Stainless Steel in the Ultrasonic Bending Test

**DOI:** 10.3390/ma17071657

**Published:** 2024-04-04

**Authors:** Yongtao Hu, Sen Tang, Yongjie Liu, Lang Li, Chong Wang, Qingyuan Wang

**Affiliations:** 1 Failure Mechanics and Engineering Disaster Prevention and Mitigation Key Laboratory of Sichuan Province, Sichuan University, Chengdu 610207, China; yongthu@163.com (Y.H.); liuyongjie@scu.edu.cn (Y.L.); lilang@scu.edu.cn (L.L.); wangqy@scu.edu.cn (Q.W.); 2 MOE Key Laboratory of Deep Earth Science and Engineering, College of Architecture and Environment, Sichuan University, Chengdu 610065, China; 3 School of Architecture and Civil Engineering, Chengdu University, Chengdu 610106, China

**Keywords:** ultrasonic fatigue testing, flexure fatigue, stress gradient, internal failure, nanoindentation

## Abstract

Nanoindentation measurements were conducted to investigate the high-cycle response of 316L stainless steel in bending fatigue. Hardness variation owing to the gradient flexure stress amplitude for different curvatures was plotted along with the thickness and length, respectively. Scanning electron microscopy (SEM) was subsequently conducted to explore the deformation characteristics in multiple layers, which had cyclic gradient stress, on the cross-section of specimens. The nanoindentation results indicated that the cyclic hardening response of 316L stainless steel is correlated with the level of stress amplitude in the high-cycle fatigue (HCF) regime. Furthermore, an analytical model was proposed to clarify the relationship between nanohardness and stress amplitude. Finally, the evolution of damage accumulation due to irreversible plastic deformation is continuous during stress reduction up to the neighboring zone at the neutral surface of the flexure beam in some individual grains.

## 1. Introduction

316L austenitic stainless steel serves as a crucial structural material in nuclear power plants, mainly as the primary circuit main pipeline in a pressurized water reactor [1], owing to its excellent corrosion [2] and heat resistance [3]. The main pipeline is subjected to alternate vibration stress caused by flow impact [4] for up to 10^7^ cycles and even beyond. Therefore, understanding the cyclic behavior of 316L stainless steel under alternating vibration is essential for designing fatigue resistance.

The cyclic behavior (cyclic hardening or softening) of materials is directly influenced by loading cycles and stress amplitudes during fatigue loads. For the low-cycle fatigue (LCF) condition, the cyclic response of 316L stainless steel under uniaxial tension-compression (TC) loading has been extensively studied [5,6,7,8,9,10]. The material exhibits continuous cyclic hardening in a short loading cycle, followed by cyclic softening until fatigue failure occurs [7,8,10]. The effect of strain amplitude on its cyclic response lies in its hardening or softening rate, i.e., the increase or decrease rate of normal maximum stress in the LCF regime [11]. Lower strain amplitudes result in a lower initial hardening rate [9,10]. A similar hardening rate response was observed in the study of TC consecutive deformation behavior in Mn18Cr18N austenitic stainless steel [12].

The deformation mechanism of the microstructure is closely related to the cyclic hardening and softening process. In the LCF regime, the cyclic hardening or softening of 316L stainless steel is associated with the dislocation density of the microstructure, i.e., dense, tangled, and evenly distributed dislocation leads to cyclic hardening [6,7]. Conversely, dislocation rearrangement into stable structures, such as labyrinth, wall, and channel structures, results in cyclic softening [5,6]. Regular structures are typically presented as intensely active persistent slip bands (PSBs) on the specimen surface during this phase [13]. For HCF and very high-cycle fatigue (VHCF) conditions, the material exhibits a fatigue limit, allowing the formation of non-propagating microcracks at surface slip bands due to α’-martensite formation near the crack tip [14]. However, for 316L stainless steel, limited martensite formation is observed only in areas closely related to long crack growth at room temperature [15,16,17].

To date, researchers have also conducted many studies on the high- and very high-cycle cyclic response of 316L stainless steel under ultra-high frequency loading. Pu et al. [18] showed that fatigue loading increases the yield strength of the material and that cyclic hardening may be the main response behavior under high-cycle fatigue. Xiong et al. [19] showed that 316L stainless steel exhibited a cyclic hardening phenomenon in the VHCF regime and its microhardness increased with increasing cycle time. Fu et al. [20] characterized the cyclic response of austenitic steel under varying high-cycle fatigue by introducing nanoindentation, and the results showed that the material first undergoes cyclic softening, then cyclic hardening, and finally the cyclic hardening phenomenon. Grigorescu et al. [14] characterized the cyclic response of 316L stainless steel in the VHCF regime by measurement of changes in resonant frequency. The results showed that at high-stress amplitudes, cyclic softening occurs first, followed by cyclic hardening. By contrast, when the stress amplitude is approximately equal to the fatigue limit, only cyclic hardening is observed. However, 316L stainless steel is also challenged by continuously changing stress levels in service, so the cyclic response of 316L due to changing stress levels under ultra-high frequency loading also needs to be further investigated.

In this study, bending fatigue loading of 316L was carried out by an ultrasonic fatigue loading system to ensure the loading conditions of gradient stress and ultra-high frequency rate. The surface and cross-section of the specimens were tested using a nanoindentation measurement technique to investigate the effect of stress amplitude and stress gradient on the material at ultra-high loading frequency. Finally, the microstructure was analyzed using scanning electron microscopy to illustrate the plastic deformation transition mechanism.

## 2. Experiment Procedures

**Figure 1:**
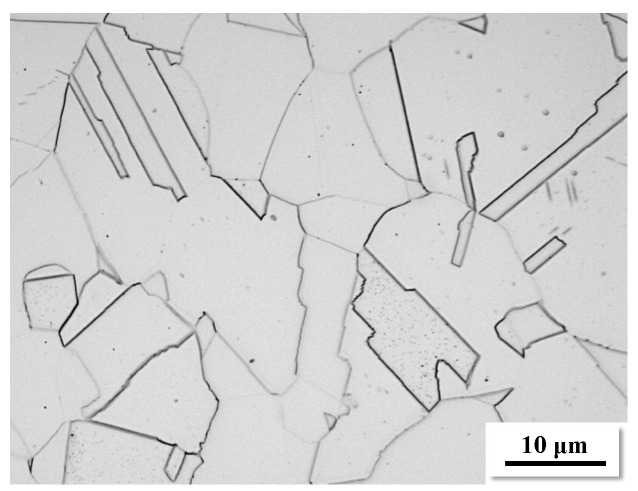
Microstructure of 316L stainless steel by optical microscopy (OM).

The material examined in the present study was 316L stainless steel subjected to solution annealing treatment at 1050 °C for 30 min. The metallographic sample was prepared using standard grinding and polishing techniques. Etching was performed by immersing the sample in a corrosion solution (with a ratio of HNO_3_:HCl:H_2_O = 1:10:10) for 2 to 3 min, followed by rinsing with deionized water. Fatigue specimens were mechanically polished using a grinding and polishing machine to achieve a flat surface, then electrolytically polished with a mixture of 10% perchloric acid and 90% alcohol (by volume) to reduce the residual stress introduced by mechanical polishing. Figure 1 illustrates the microstructure of 316L stainless steel obtained with an optical microscope, revealing typical austenitic grains with twinning structures, and the average roughness of this area is 0.3 mm. The monotonic mechanical properties are indicated in Table 1.

**Figure 2:**
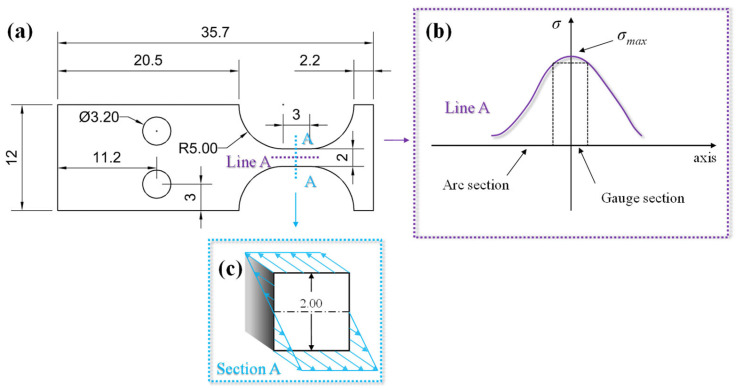
The size and stress distribution of specimens in the ultrasonic bending fatigue tests (dimensions in mm). (**a**) The sketch of specimens. (**b**) The stress distribution on the surface along the axis. (**c**) The stress gradient distribution in Section A.

Figure 2a depicts the specimen dimensions, with a thickness of 2 ± 0.02 mm. The stress distribution in the gauge section on the surface is illustrated as Line A in Figure 2b, with *σ_max_* denoting the maximum loading stress. Because stress gradients were present on the gauge section, the stress amplitude was generally considered to be equivalent to *σ_max_* in the fatigue tests. Additionally, the stress gradient on the cross-section is depicted in Figure 2c. Therefore, during fatigue loading, the stress on the surface of the specimen showed a continuous distribution, while in the hazardous section, the stress showed a gradient distribution.

**Figure 3:**
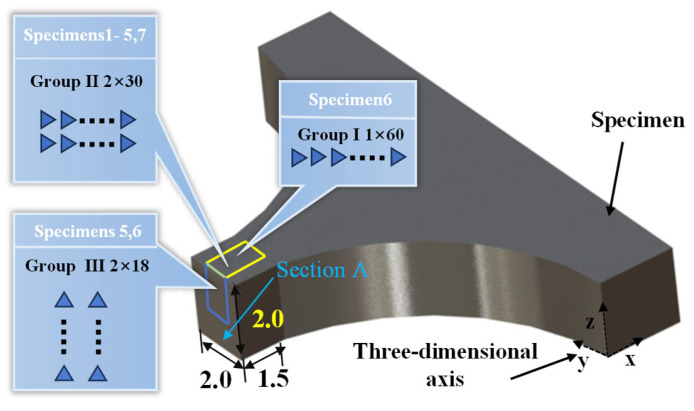
The detailed distribution of indentations (dimensions in mm).

Nanoindentation tests (KLA iNano, Milpitas, CA, USA) were directly performed on the surface (during the fatigue test) and cross-section (at the end of the fatigue test) of the specimens at room temperature. In addition, since the stresses on the specimen scalar segments showed a symmetrical distribution (refer to Figure 2), it was possible to divide the test area into three groups (I, II, and III), as shown in Figure 3. Due to the different stress gradients on the gauge and arc sections shown in Figure 2b, the cyclic response of the hardness values after fatigue tests varied. Therefore, 60 measurement spots were arranged within one column on the surface for specimen No. 6, as depicted by Group I in Figure 3. The alternating arrangement of two columns was positioned only on the gauge area for other specimens, as shown by Group II. Due to the symmetrical stress distribution on the cross-section shown in Figure 2c, the indentation matrix of Group III consisted of 36 indentation points with two rows and 18 columns. Each indentation point was 50 μm apart for all three groups. The nanoindentation tests were performed at the same strain rate at 0.2 s^−1^. The maximum loading force, displacement, and holding time were 25 mN, 5000 nm, and 1 s, respectively. The nanoindentation hardness was finally converted to Vickers hardness by formula calculation [21].

**Figure 4:**
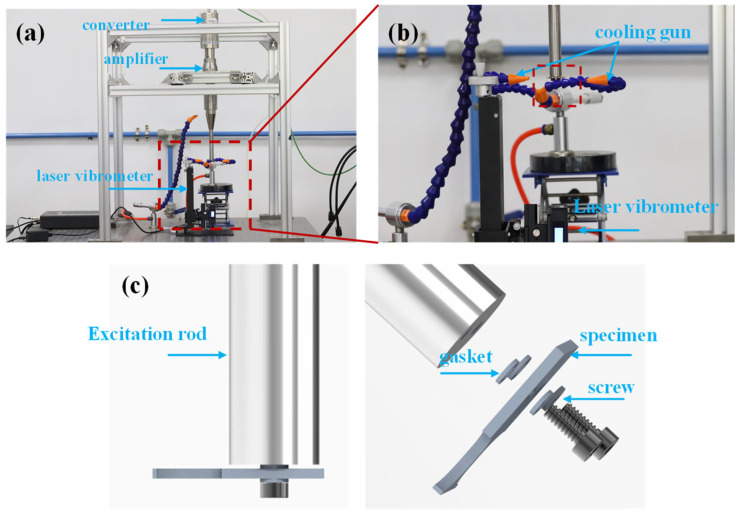
Ultrasonic bending fatigue system [22]: (**a**) full view of the system; (**b**) details of the constraint; (**c**) specimen installation diagram.

An ultrasonic bending fatigue system with a loading frequency of 20 kHz and a stress ratio of R = −1 was used in this experiment [22], as shown in Figure 4. The test loading direction was along the Z-axis, as shown in Figure 3. The test required a pause at 5 × 10^4^, 10^5^, 5 × 10^5^, 10^6^, and 5 × 10^6^ cycles for nanoindentation characterization, and the test was considered to be completed when the cycle number reached 10^7^ or fatigue fracture occurred. Intermittent loading mode (110 ms of loading time followed by 800 ms of rest time) and cooled compressed air were employed to mitigate thermal effects during testing.

Optical microscopy (OM, OLYMPUS GX53, Tokyo, Japan) and scanning electron microscopy (SEM, JEOL6510LV, Tokyo, Japan) were utilized to analyze the morphology of the surface and cross-section of specimens.

## 3. Results

### 3.1. Hardness Responses with Cycles on the Surface

**Figure 5:**
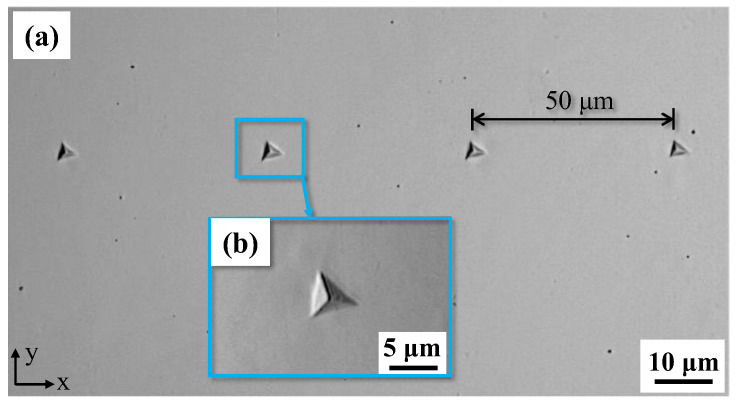
(**a**) Optical microscope image of four nanoindentations points after electrolytic polishing. (**b**) An enlarged image of an indentation.

Figure 5a presents OM images of the specimen surface after conducting nanoindentation tests. The indentations were carried out within the matrix shown by Group I in Figure 3. Figure 5b depicts an enlarged image of an indentation within the matrix.

In the present study, seven specimens were tested with stress levels ranging from 320 MPa to 350 MPa, as detailed in Table 2. Specimen 7 experienced fatigue failure after 6.5 × 10^5^ cycles at a stress amplitude of 350 MPa. Fatigue tests for the other specimens were terminated upon exceeding 1 × 10^7^ cycles. The average hardness values from the gauge section tests are shown in Table 2 for the beginning and the end of the fatigue test. The average hardness of 316L stainless steel after cyclic loading increased as fatigue life accumulated up to the HCF regime. A similar evolutionary principle was observed for specimen 7, which experienced fatigue failure at 350 MPa. This suggested that the surface mechanical properties (yield stress) of the specimens can be enhanced with the accumulation of cyclic bending loading, consistent with findings in the reference work [18].

**Figure 6:**
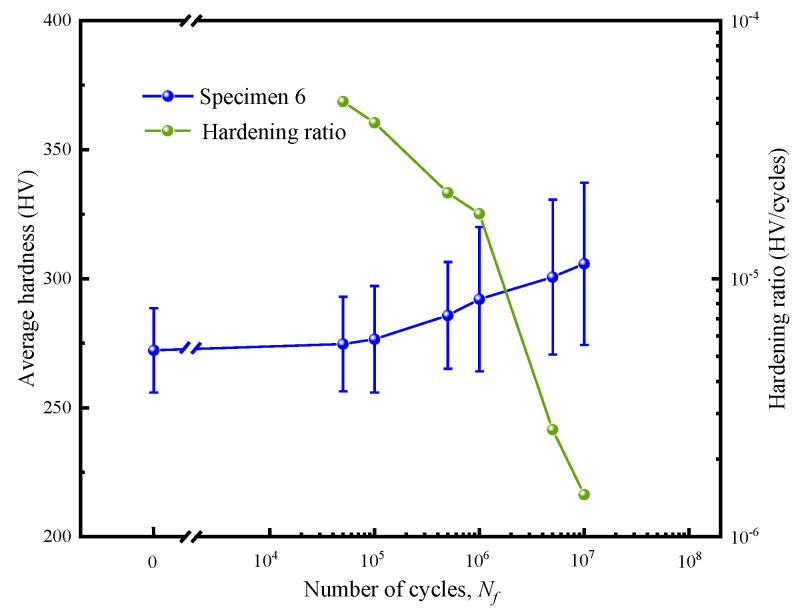
The average hardness of specimen No. 6 changes with the number of cycles, as shown in blue spherical points, and the cyclic hardening rate is shown in green spherical points.

The blue spherical polyline in Figure 6 depicts the average hardness obtained at spots in Group I on the surface of specimen 6 in response to the number of cycles. It shows that hardness increased gradually with the rise in cycle number and illustrates that cyclic hardening of this material did occur. The green spherical polyline in Figure 6 shows the cyclic hardening ratio (HR) in different stages. The parameter HR is defined as follows:

(1) $$\mathrm{HR}=\frac{\Delta V}{N_{f}}=\frac{V-V_{i}}{N_{f}}$$

where *V* represents the hardness at the current test and *V_i_* represents the hardness of the previous test. *N_f_* represents the number of cycles. The cycle hardening rate was the largest initially and gradually decreased in the subsequent stages, but the value was always greater than zero. This indicated that cycle hardening existed in the whole fatigue failure process.

### 3.2. Hardness Response with Stress Distribution on the Surface

**Figure 7:**
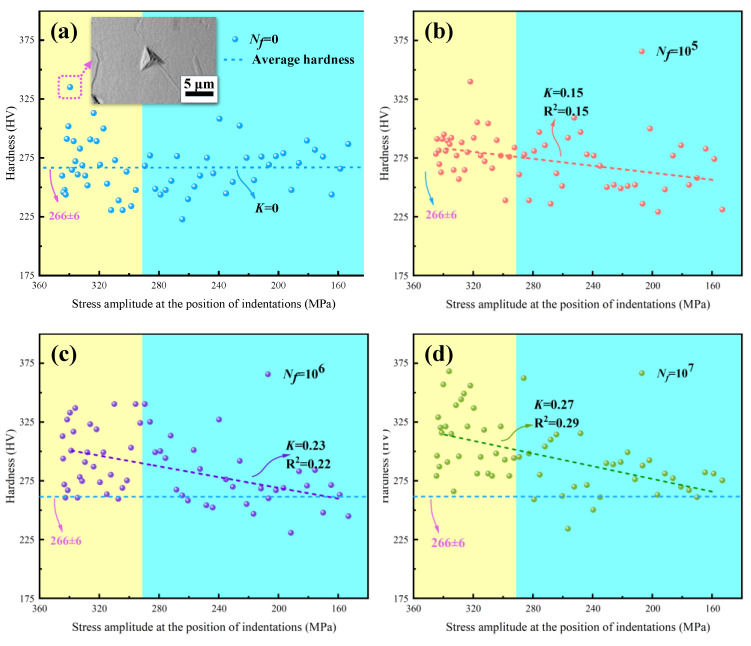
(**a**–**d**) Hardness on the surface of specimen No. 6 changes with stress amplitude at different numbers of cycles.

Figure 7 depicts the hardness responses along with stress distribution on the surface of specimen 6, where the maximum stress was located at the section with the smallest area on the gauge section and the minimum stress was located in the area with the connecting arc segment (refer to Figure 2b). Blue, red, purple, and green spherical points represent hardness values under different number of cycles from 0, 10^5^, 10^6^, to 10^7^, respectively. The dotted lines in Figure 7 represent linear fitting curves of the corresponding data, with their slopes denoted as K. The blue dotted lines perpendicular to the vertical axis represents the initial average hardness value (266 ± 6 HV, within a 95% confidence interval) of specimen 6. The blue spherical points fluctuate around the blue dotted line, and it is worth noting that a significant increase in the hardness value occurred when the indentation point was located on a grain boundary, as shown in the dashed box of Figure 7. The study by Voyiadjis et al. [23] also demonstrated that grain boundaries lead to the hardening phenomenon during nanoindentation of FCC metals [23]. With the increase in the number of cycles, the significant dispersion of hardness values, as shown in Figure 7b–d, indicated inhomogeneous deformation of the microstructure [24,25,26].

Furthermore, the horizontal axis was divided into yellow and blue regions, based on the transitions of indentation points from the gauge section to the arc section of the specimens, as depicted in Figure 3. This revealed that the hardness on the gauge section of specimen 6 was significantly higher than that on the arc section after bending fatigue tests. This discrepancy arose because the stress amplitude on the gauge section of specimens was greater than that on the arc section, as illustrated in Figure 2b. Additionally, the greater the number of cycles, the higher the value of K shown in Figure 7b–d. This further confirmed the occurrence of cyclic hardening in 316L stainless steel, aligning with the results in Figure 6.

### 3.3. Hardness Responses with Stress Gradient on the Cross-Section

**Figure 8:**
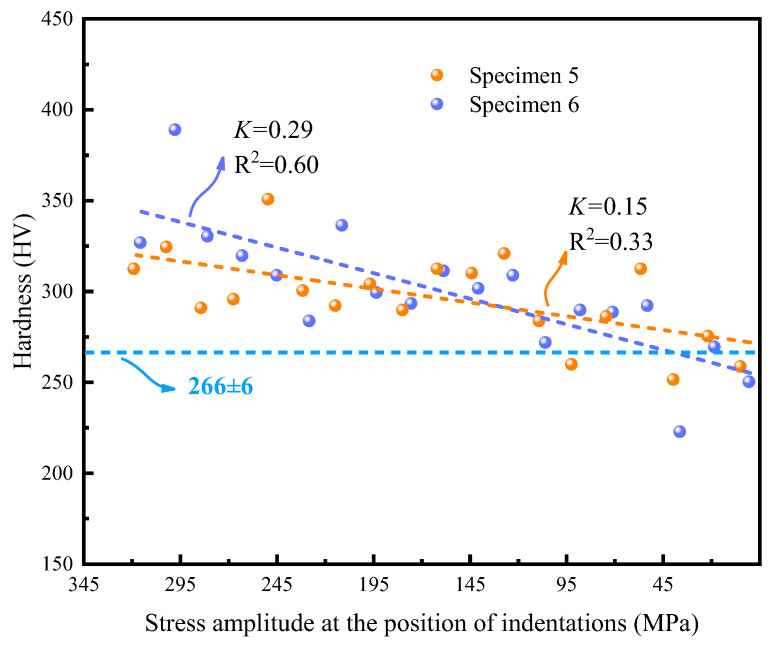
Hardness on the cross-section of the specimen changes with stress amplitude after the bending fatigue tests.

Figure 8 shows the hardness distribution of specimens 5 and 6 on the cross-section at the same loading stress amplitude and number of cycles (i.e., *N_f_* = 1 × 10^7^, *σ_a_* = 345 MPa). The hardness results of the two specimens are represented by the orange and blue spherical points, respectively, with corresponding linear fitting curves depicted in dotted lines. The blue dotted line also indicates the initial average hardness. Due to the symmetrical distribution of the stress amplitude along the thickness of the specimens, as shown in Figure 2c, only the half-thickness of the cross-section needed to be measured by nanoindentation tests. Theoretically, the maximum value of stress was 345 MPa at the surface, and the minimum was zero at the center of Section A.

Figure 8 also shows that the hardness value gradually decreased with decreasing stress level, which was the same as the results in Figure 7. The hardness near the surface was observed to be higher than that at the neutral layer of the cross-section. The reason is that the stress was the largest near the surface and the smallest near the center. Note that the slopes of the two curves are different with the same loading stress and cycles. This may have been caused by the slight difference in thickness and microstructure distribution of the specimens. Moreover, the discrete distribution of the hardness indicated the microstructural sensitivity. This was similar as to why the surface hardness values of the specimens showed great dispersion after the fatigue tests in Figure 7a–c.

### 3.4. Deformation of Microstructures in the Cross-Section

**Figure 9:**
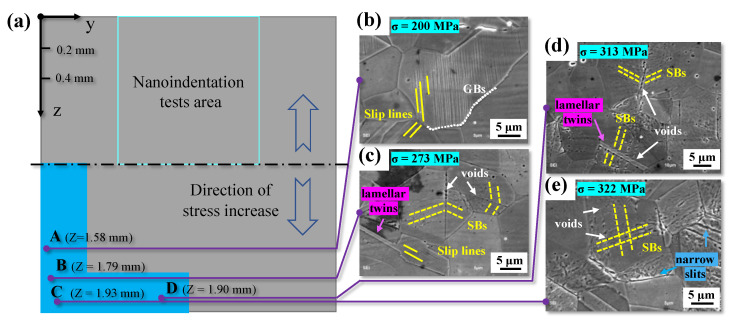
Microstructure in the cross-section of specimen 6 (*σ_a_* = 345 MPa, *N*_f_ = 1 × 10^7^). (**a**) Sketch of the cross-section with a length of 2 mm on the YZ region (the blue area was carefully observed by SEM); (**b**–**e**) SEM images of positions A, B, C, and D, respectively.

The positive stress (σ) in the cross-section of a beam under pure bending conditions satisfies the following relationship:

(2) $$\sigma =-\frac{M_{z}}{I_{z}}y$$

where *E*, *I_z_*, and *M_z_* represent the modulus of elasticity of the material, the moment of inertia to the Z-axis, and the bending moment, respectively. It can be seen that the positive stresses show a linear distribution in the cross-section (refer to Figure 2c). Therefore, according to the establishment of the plane coordinate system shown in Figure 8, it is known that the stress (*σ_z_*) on any thickness can be expressed as follows:

(3) $$\sigma_{\left(z\right)}=\sigma_{surface}-\frac{\sigma_{surface}}{t}z$$

where *σ_surface_*, *t*, and *z* are the maximum positive stress on the surface of the specimen, the specimen’s half-thickness, and the depth from the surface, respectively. Figure 9 shows the microstructure in the cross-section of specimen 6, which was cut in two parts following a slight polishing and etching. Figure 9a shows a cross-section sketch of the YZ region in Figure 3. In the present study, the blue area was observed by SEM for different stress levels, as shown in Figure 9a. The magnified images of four positions (A, B, C, and D in Figure 9a) in the blue area are shown in Figure 9b, Figure 9c, Figure 9e and Figure 9d, respectively. The stress levels of the four locations were 200 MPa, 273 MPa, 322 MPa, and 313 MPa, determined by Equation (3).

In some grains, regular linear marks left by single system slip lines could be observed, as depicted by the solid yellow lines in Figure 9b. It is worth noting that the slip motion occurred only within the grain, as the grain boundaries of adjacent grains with slip lines remained intact, as indicated by the white dotted line in Figure 9b. However, rows of gully-like slip bands emerged in the majority of grains at position B, as illustrated by the yellow dotted lines in Figure 9c. Simultaneously, a few grains in Figure 9c also exhibited the regular linear marks mentioned earlier. At the junction of slip bands in grain or twin boundaries, irregular spherical voids (white arrows in Figure 9c) became visible.

At positions C and D near the surface, dense gully-like slip bands were visible in most grains, as depicted in Figure 9d,e. Irregular spherical voids were also observed at the boundaries of lamellar twins (bright purple arrows in Figure 9c,d), except at the grain boundaries mentioned earlier in Figure 9c. Additionally, dense slip band marks in grains with slightly etched pits on the slip bands are evident in Figure 9d. Furthermore, a multi-system of slip bands (yellow dotted line in Figure 9e) was discernible in some grains. Interestingly, irregular spherical voids could also be observed at the intersection of multiple slip bands, as indicated by the white arrows in Figure 9e. Moreover, numerous narrow slits (blue arrows in Figure 9e) were etched at the positions of grain and twin boundaries, intersecting precisely with the straight direction of slip bands.

## 4. Discussion

### 4.1. The Influence of Stress Amplitude on the Hardness

**Figure 10:**
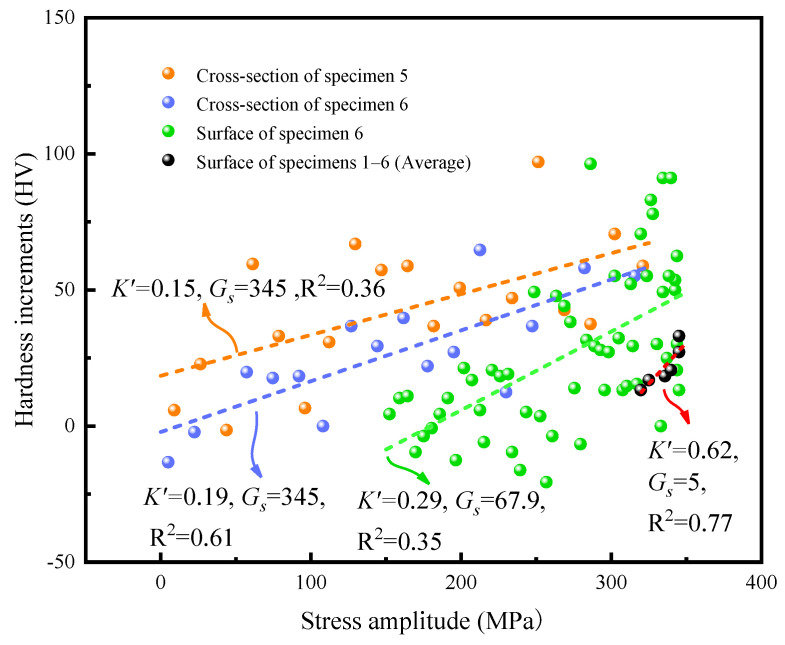
The relationship between stress amplitude and the hardness of 316L stainless steel.

Figure 10 presents the correlation between hardness increments and stress amplitude in the high-cycle bending fatigue regime. Dark blue and green spherical points represent the distribution of hardness with stress amplitude on the surface and the cross-section of specimen 6, respectively. Orange spherical points depict the hardness distribution on the cross-section of specimen 5. It is noteworthy that the initial average hardness value is subtracted to mitigate the influence of different specimens on the results, resulting in some hardness increments being negative. Additionally, black spherical points indicate the difference in hardness at the beginning and end of fatigue tests among specimens 1–6, with corresponding dotted lines representing linear fitting curves of these data. K′ represents the slope of the variation of hardness with stress amplitude.

More importantly, the gradient of different stress distribution types is expressed by Gs, which represents the stress gradient interval at the test point in the cross-section. It was easier to obtain the stress gradient on the cross-section of specimens 5 and 6 as 345 MPa/mm. The stress on the surface followed a parabolic distribution, as depicted in Figure 2b, and was then fitted with a linear curve to approximate the stress gradient. Therefore, the stress gradients on the surface of specimen 6 were calculated to be 67.9 MPa/mm. It is important to note that the varying loading stress on each specimen was reasonably considered to be equivalent to the stress amplitude distributions at different positions of one specimen, resulting in a stress gradient of 5 MPa/mm (depicted as black points).

Figure 10 illustrates that the increment in hardness decreases with a decrease in stress amplitude, indicating the high-strain hardening characteristic of 316L stainless steel, even under different stress gradients [27]. Since the specimen was in a bending stress state, its stress varied continuously along the specimen surface and showed a gradient distribution along the cross-section [28,29,30]. It was easy to conclude a general rule through a large number of hardness data.

The greater the stress gradient, the smaller the value of K′, as shown in Figure 10. This indicates that the change rate of hardness increment to the stress amplitude exhibits an inversely proportional relation to the stress gradient. For a given maximum stress amplitude, a higher stress gradient leads to a smaller nominal stress amplitude and, consequently, a smaller increase in hardness.

As shown in the above results in Figure 6 and Figure 7, the increase in average hardness on the surface of 316L stainless steel with an increase in the number of cycles indicates that only cyclic hardening occurs in the HCF regime [31]. This is different from the deformation characteristics in the LCF regime, i.e., cyclic hardening occurred first in fewer cycles, and then cyclic softening occurred until failure [7]. However, the number of cycles and the stress amplitude play similar roles in the hardness increase of 316L stainless steel, as shown in Figure 6 and Figure 10. It should be noted that the influence of stress amplitude on cyclic hardening is the most critical. The effect of stress amplitude on hardness is mainly reflected by the microstructural response to the stress amplitude, but the number of cycles only promotes this response process.

### 4.2. Deformation Characteristics in Gradient Stress

**Figure 11:**
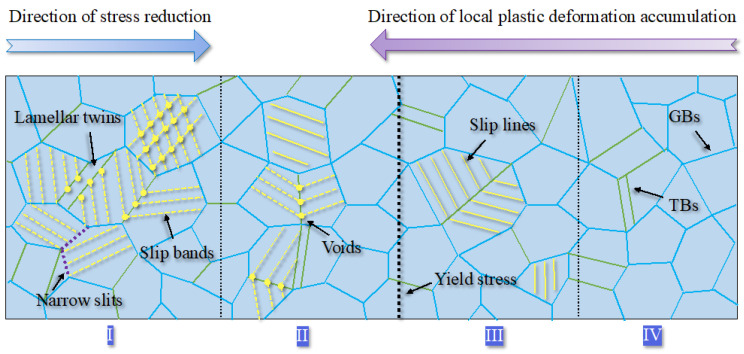
Collaborative deformation of 316L stainless steel under gradient stress.

It is widely recognized that fatigue loads results in localized damage accumulation in the microstructure. However, the microstructural response has yet to be fully understood under cyclic gradient stress. Figure 11 shows the sketch of the collaborative deformation characteristics of the microstructure under bending fatigue tests. The higher the stress amplitude, the more obvious the corresponding plastic deformation accumulation [32]. Blue solid, green, and black dotted lines represent grain boundaries, twin boundaries, and regional boundaries, respectively. The boundaries between each region (from I to IV) in Figure 11 are artificially included to correspond with the SEM results in Figure 9.

Region I in Figure 11 represents the bulk of the material on the surface, where the stress level induces maximum deformation in the material. Grains with different orientations, sizes, and hardness are deformed differently to maintain the continuity of the microstructure. Elastic-plastic deformation occurs in the majority of grains, while, on the contrary, only elastic deformation occurs in some grains with unfavorable slip orientation. Furthermore, irreversible plastic deformation of 316L stainless steel likely leads to crystal slip motion and forms slip bands at room temperature in the HCF regime [33,34]. The closed-packed planes of 12 slip systems in face-centered-cubic (FCC) crystals are {111} planes and also called favorable slip planes [35], where single slip occurs first and then multi slip caused by the continual accumulation of local plasticity during the fatigue tests occurs [36,37]. Region I in Figure 11 also illustrates a deformation behavior, i.e., multi-slip bands intersect in some grains while single-slip bands meet at the boundaries of most grains, forming voids at the intersection. For most grains in Figure 9, dislocations driven by the critical shear stress accumulate first at the grain and twin boundaries and then form a random arrangement of dislocations in low strain amplitude while an organized structure such as a cellular structure in high strain amplitudes, respectively [5,38,39]. Finally, the slip bands observed by SEM shown in Figure 9 comprise uniform dislocation structures [13].

Region II in Figure 11 corresponds to the SEM image of position B shown in Figure 9c, where the accumulation of local plastic strains is insufficient to form multi-slip bands in grains as the stress amplitude reduces. The patterns indicate that single slip bands in adjacent grains mostly meet at the twin boundaries. A few are inside grains without penetrating the grain boundaries, as depicted in Figure 9b. The bold dotted line between Regions II and III in Figure 11 represents the location where localized stress equals the nominal yield strength (220 MPa) of 316L stainless steel.

Observing position A (200 MPa) shown in Figure 9b, Region III in Figure 11 indicates that irreversible local plastic deformation (slip lines) persists even when the stress amplitude is lower than the yield strength of the material. This reveals strong evidence of localized damage for the internal crack initiation at low-stress amplitude in the VHCF regime [40,41,42]. Finally, the microstructure in Region IV indicates no prominent plastic deformation, same as the initial results shown in Figure 1.

The formation mechanism of voids indicated by yellow solid points in Figure 11 primarily arises from the creation and destruction of numerous vacancies, leading to the formation of microdefects in slip bands and grain boundaries during the cyclic deformation process [43,44,45]. Another factor is the higher distortion energy at the intersection of grain or twin boundaries and slip bands due to the accumulation of dislocations [46], causing the microdefects to corrode more easily into spherical or ellipsoidal voids, as shown in Figure 9c–e. The narrow slits are likely formed by combining enough small voids at the grain boundary and further developing into micro cracks in the subsequent cyclic loading.

In summary, the cyclic response of plastic deformation in the direction of the stress increment progresses continuously from light slip lines to multi-slip bands due to the stress gradient.

## 5. Conclusions

The cycling behavior of 316L stainless steel under the HCF condition was investigated by nanoindentation tests. The main conclusions are as follows:

(1) Under high-cycle bending fatigue loading, a cyclic hardening behavior was found for 316L stainless steel. As the number of cycles increases, the hardening rate increases and the change in hardening rate decreases.
(2) The greater the stress amplitude, the greater the increase in hardness, whereas the greater the stress gradient, the smaller the increase in hardness.
(3) As the stress amplitude increases, the mode of plastic deformation within the grain changes from single slip to cross slip.

## Figures and Tables

**Table 1:** Mechanical properties of 316L stainless steel [4].

Mechanical Properties	Value
Yield Stress (0.02%; MPa)	220
Tensile Strength (MPa)	580
Density (kg/m^3^)	7990
Young’s Modulus (GPa)	195

**Table 2:** The results of bending fatigue tests and nanoindentation tests.

Specimen Number	Bending Fatigue Tests		Nanoindentation Tests	Average Hardness on the Surface (HV)		
	Stress (*σ_a_*, MPa)	*N* (Cycles)	Arrangement Groups	Beginning	End	Difference
1	320	1 × 10^7^ (stop)	II	322.4	335.7	+13.3
2	325	1 × 10^7^ (stop)	II	305.6	322.6	+17.0
3	335	1 × 10^7^ (stop)	II	304.6	323.5	+18.9
4	340	1 × 10^7^ (stop)	II	283.3	304.3	+21.0
5	345	1 × 10^7^ (stop)	II, III	253.7	281.0	+27.3
6	345	1 × 10^7^ (stop)	I, III	272.0	305.1	+33.1
7	350	6.5 × 10^5^ (rupture)	II	288.3	303.9	+15.6

## Data Availability

Data are contained within the article.
